# Supplementary material for: ATR-FTIR spectroscopy detects alterations induced by organotin(IV) carboxylates in MCF-7 cells at sub-cytotoxic/-genotoxic concentrations
Source: PMC Biophys. 2008 Nov 5;1:3. doi: 10.1186/1757-5036-1-3 (PMC2666631; doi:10.1186/1757-5036-1-3)
Supplement: Additional file 1 — SupplementaryData-PMC Biophysics Ahmad MS et al. Additional data to Ahmad et al. (2008) containing organotin(IV) carboxylates-specific effects at increasing concentrations in MCF-7 cells. [file 1757-5036-1-3-S1.pdf]

<u>Supplementary data – Ahmad *et al.* (2008)</u>

Compound no./general formula	Concentration (μg/ml)	Number of micronuclei/Binucleate MCF-7 cells (n=1,000) [one to ≥five]				
		1 MN	2 MN	3 MN	4 MN	≥5 MN
**1** **$Bu_3Sn(L1)$**	Vehicle control	96	34	17	1	1
	0.01	126	40	14	12	4
	0.1	0	0	0	0	0
	1.0	0	0	0	0	0
**2** **$BuSnCl(L1)_2$**	Vehicle control	96	34	17	1	1
	0.01	100	49	31	9	4
	0.1	126	60	38	14	6
	1.0	130	44	24	18	4
**3** **$Ph_3Sn(L1)$**	Vehicle control	96	34	17	1	1
	0.01	115	46	24	12	6
	0.1	0	0	0	0	0
	1		0	0	0	0
**4** **$Bu_2Sn(L1)_2$**	Vehicle control	106	44	15	2	0
	0.01	126	43	20	6	5
	0.1	144	32	21	3	3
	1.0	0	0	0	0	0
**5** **$Et_2Sn(L2)_2$**	Vehicle control	106	44	15	2	0
	0.01	124	34	21	3	2
	0.1	143	51	19	7	3
	1	100	53	24	5	3
**6** **$Me_2Sn(L2)_2$**	Vehicle control	106	44	15	2	0
	0.01	115	62	21	4	4
	0.1	123	70	30	12	5
	1.0	108	46	20	3	4
**7** **$Ph_3Sn(L2)$**	Vehicle control	106	44	15	2	0
	0.01	120	47	14	9	2
	0.1	88	33	14	2	2
	1.0	0	0	0	0	0
**8** **$Bu_2Sn(L2)_2$**	Vehicle control	96	34	17	1	1
	0.01	104	42	36	6	4
	0.1	130	49	18	8	4
	1.0	120	38	32	20	14
**9** **$Bu_2Sn(L3)_2$**	Vehicle control	96	34	17	1	1
	0.01	132	56	26	8	8
	0.1	118	48	20	10	4
	1.0	0	0	0	0	0

**10** **$BuSn(L3)_3$**	Vehicle control	106	44	15	2	0
	0.01	87	53	24	7	5
	0.1	100	42	18	7	4
	1.0	136	36	14	4	0
**11** **$Me_3Sn(L3)$**	Vehicle control	106	44	15	2	0
	0.01	114	58	22	12	6
	0.1	115	65	23	3	3
	1.0	158	67	22	4	4
**12** **$Et_2Sn(L3)_2$**	Vehicle control	96	34	17	1	1
	0.01	138	48	22	8	6
	0.1	136	36	8	6	4
	1.0	0	0	0	0	0

Comet-forming activity – comet tail length (CTL, μm)

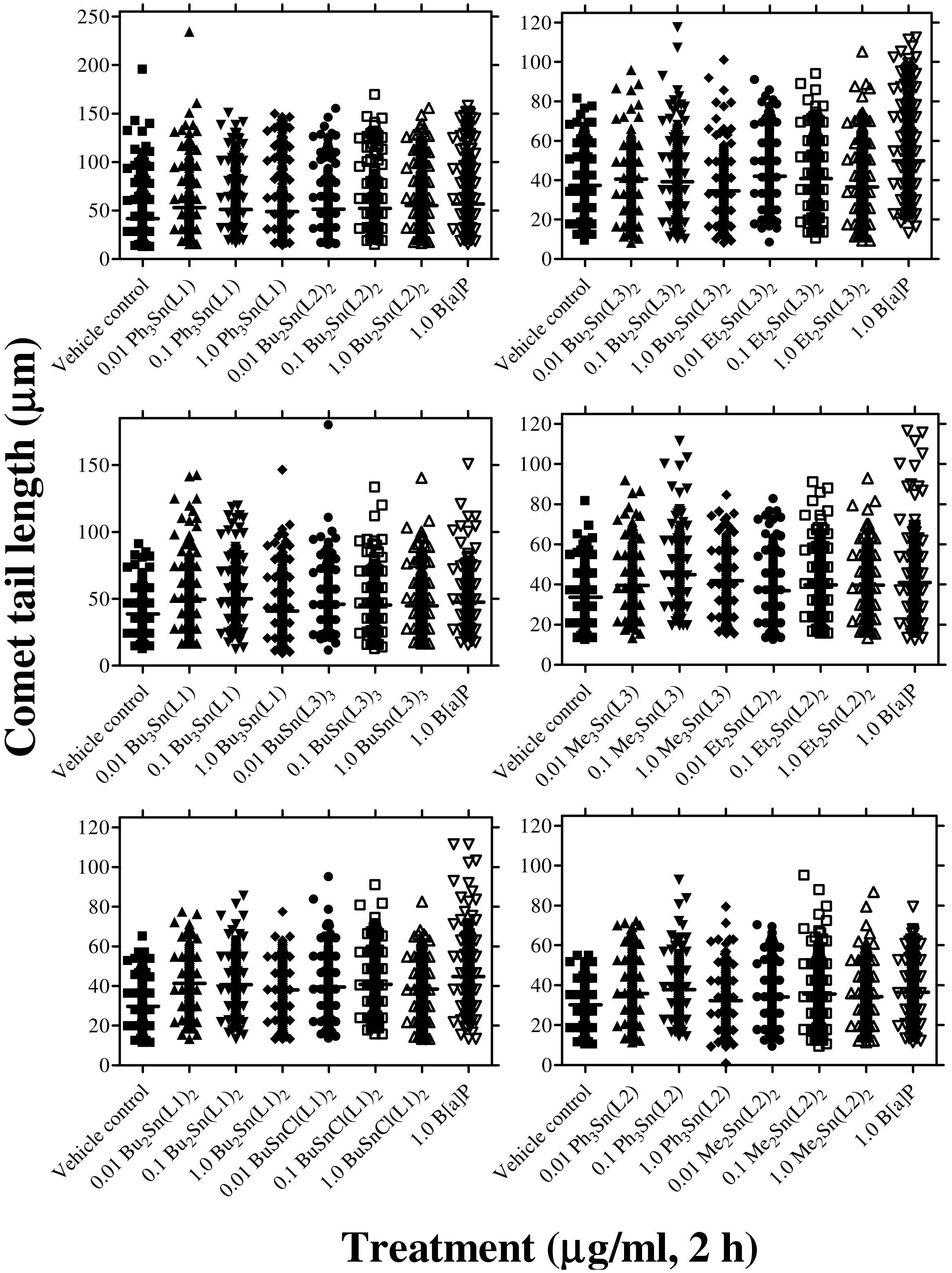

Comet-forming activity – Olive tail moment (OTM)

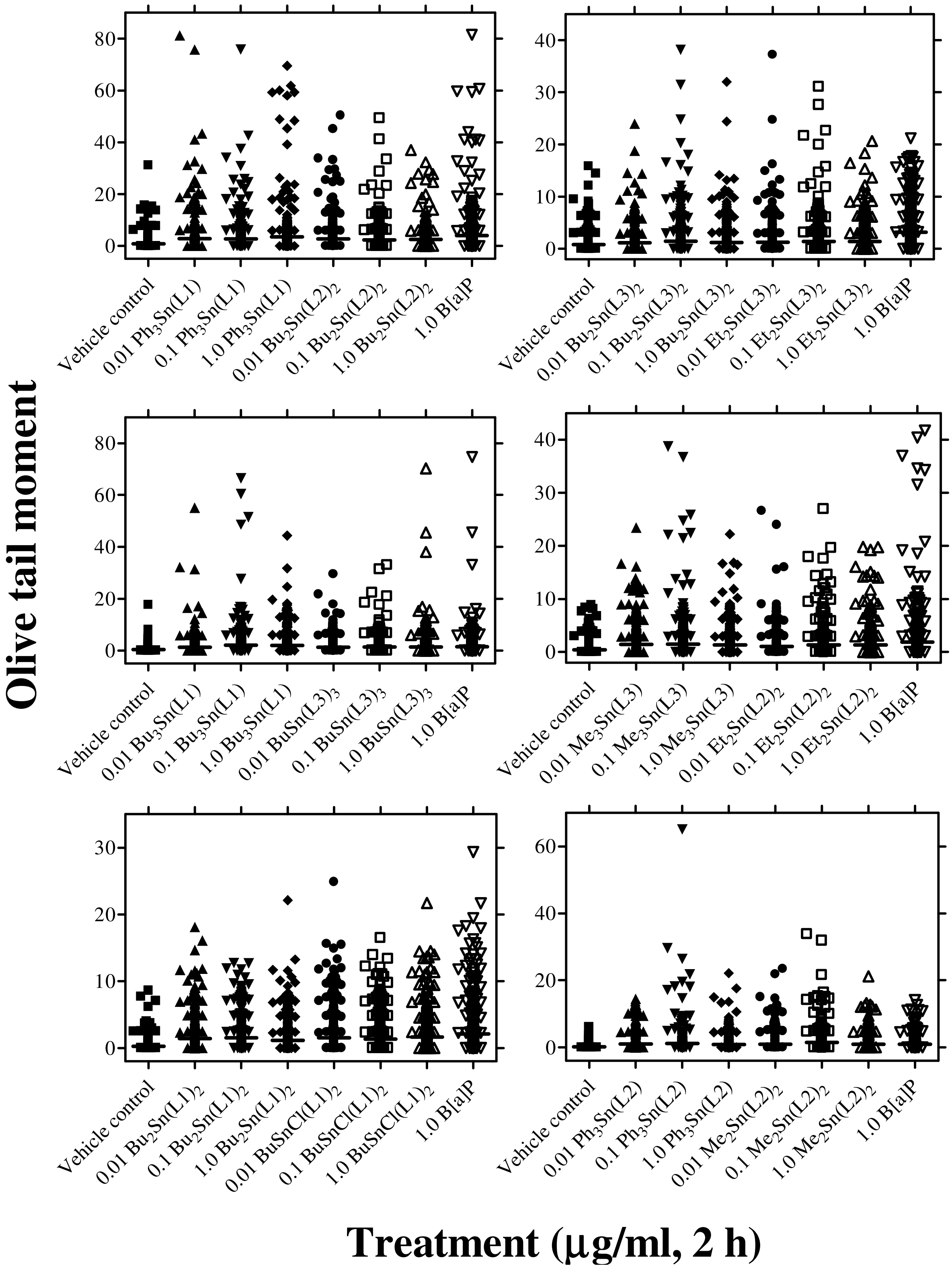

ATR FTIR spectroscopy following 24-h exposure with 0.0001 µg/ml

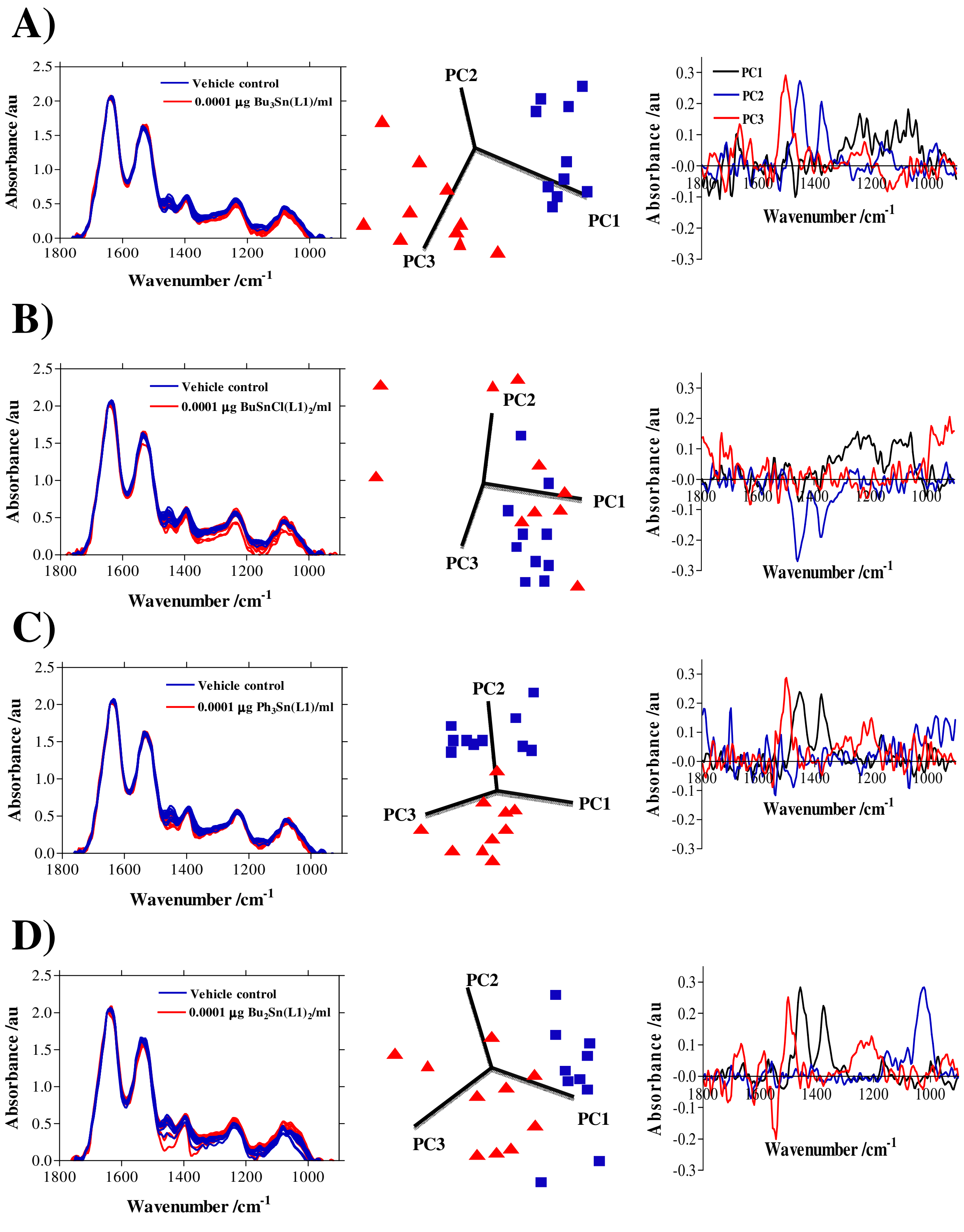

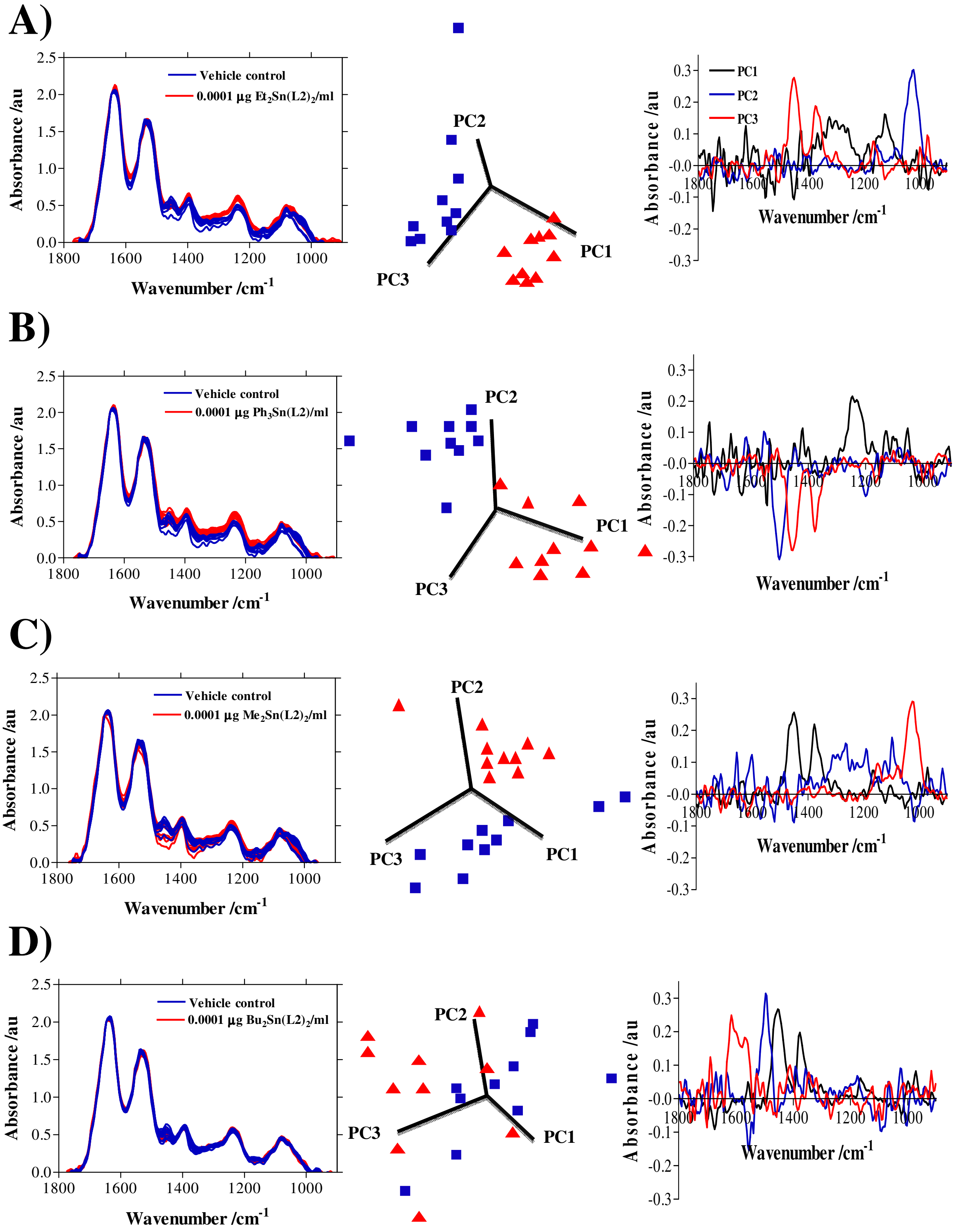
A)
Vehicle control
0.0001 µg Et2Sn(L2)2/ml
Absorbance /au
Wavenumber /cm-1
PC1
PC2
PC3
B)
Vehicle control
0.0001 µg Ph3Sn(L2)/ml
Absorbance /au
Wavenumber /cm-1
PC1
PC2
PC3
C)
Vehicle control
0.0001 µg Me2Sn(L2)2/ml
Absorbance /au
Wavenumber /cm-1
PC1
PC2
PC3
D)
Vehicle control
0.0001 µg Bu2Sn(L2)2/ml
Absorbance /au
Wavenumber /cm-1
PC1
PC2
PC3

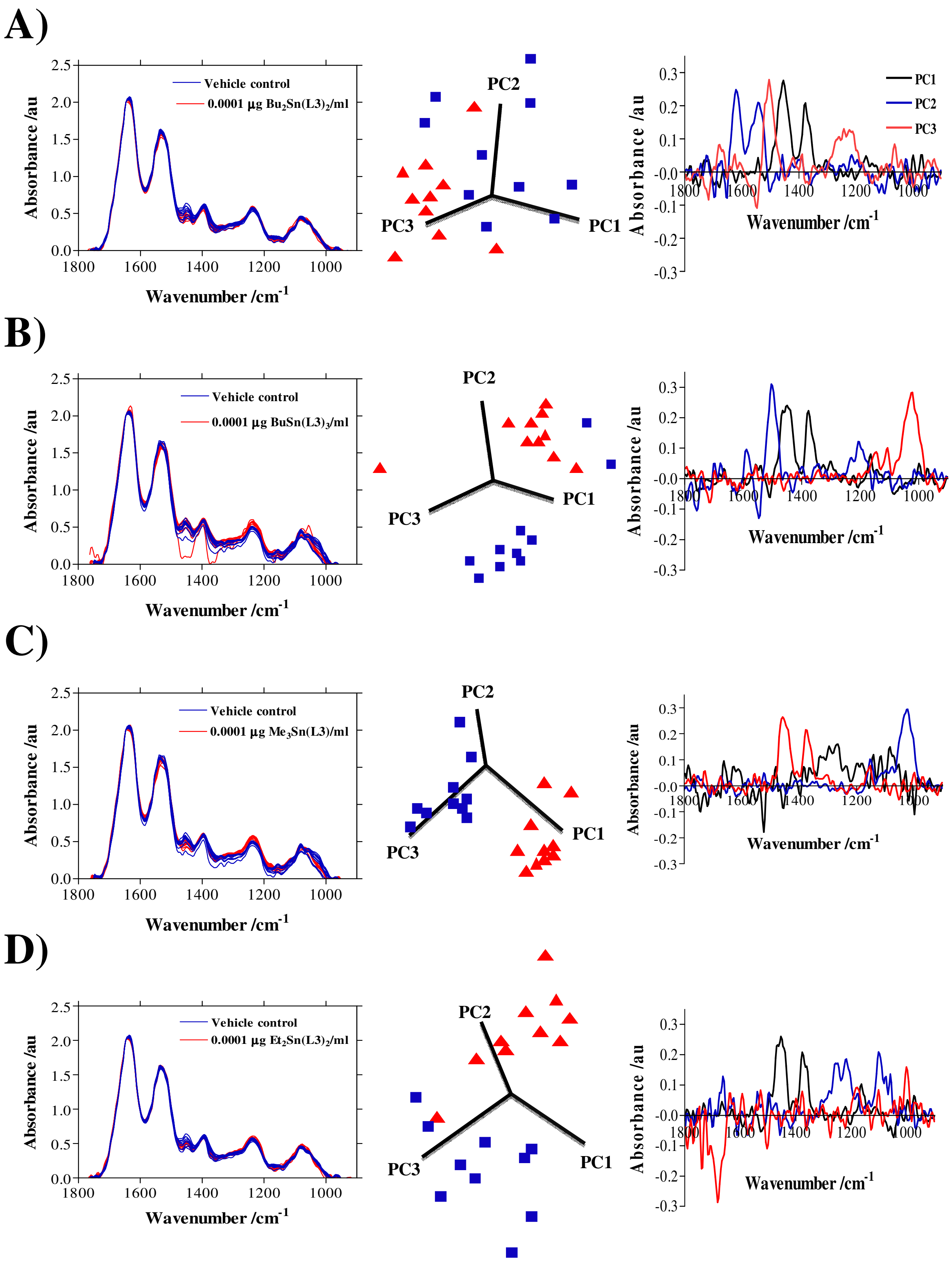
A)
B)
C)
D)
Absorbance /au
Wavenumber /cm-1
Vehicle control
0.0001 µg Bu2Sn(L3)2/ml
0.0001 µg BuSn(L3)3/ml
0.0001 µg Me3Sn(L3)/ml
0.0001 µg Et2Sn(L3)2/ml
PC1
PC2
PC3

ATR FTIR spectroscopy following 24-h exposure with 0.01 μg/ml

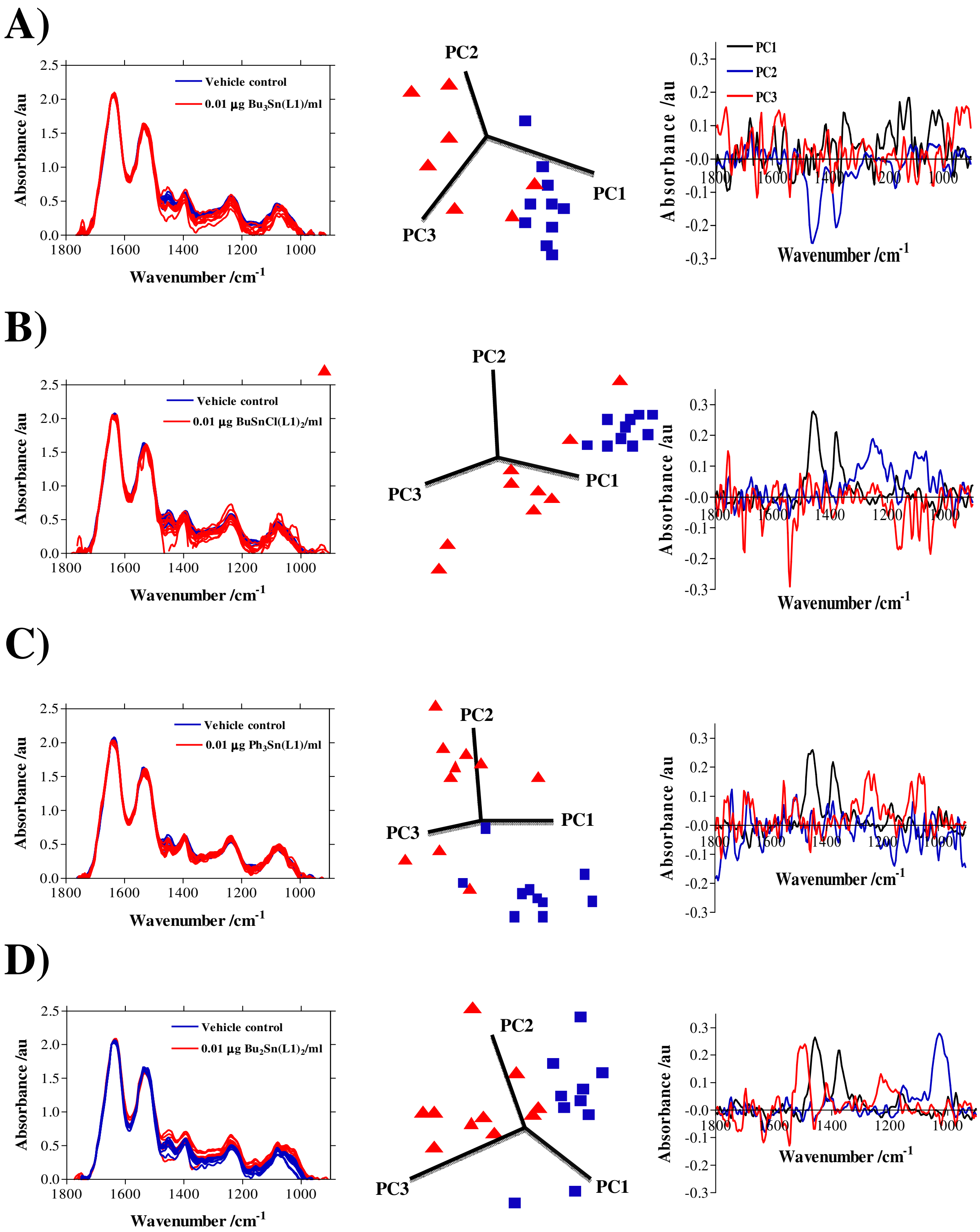

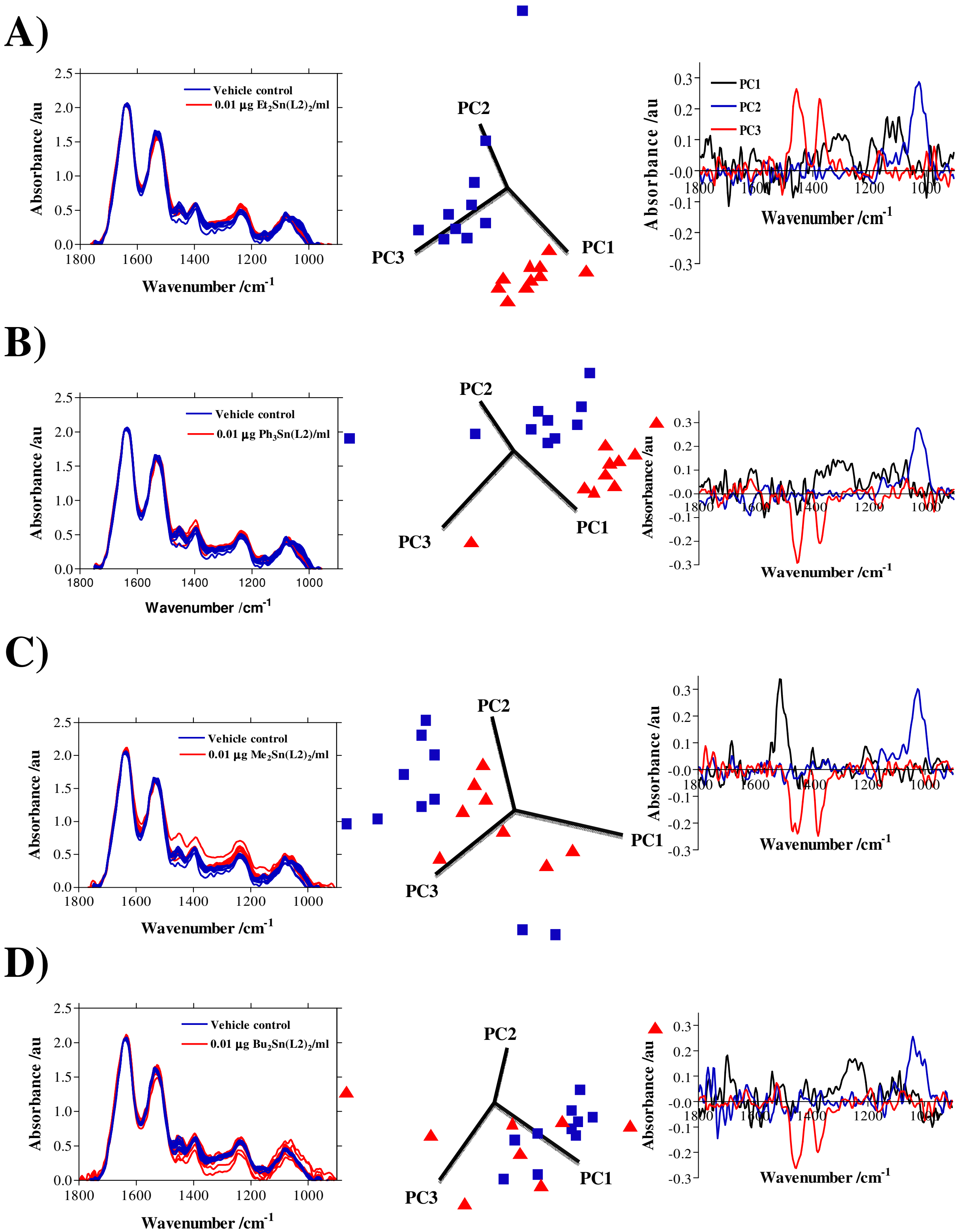
A)
Vehicle control
0.01 µg Et2Sn(L2)2/ml
Absorbance /au
Wavenumber /cm-1
PC1
PC2
PC3
B)
Vehicle control
0.01 µg Ph3Sn(L2)/ml
C)
Vehicle control
0.01 µg Me2Sn(L2)2/ml
D)
Vehicle control
0.01 µg Bu2Sn(L2)2/ml

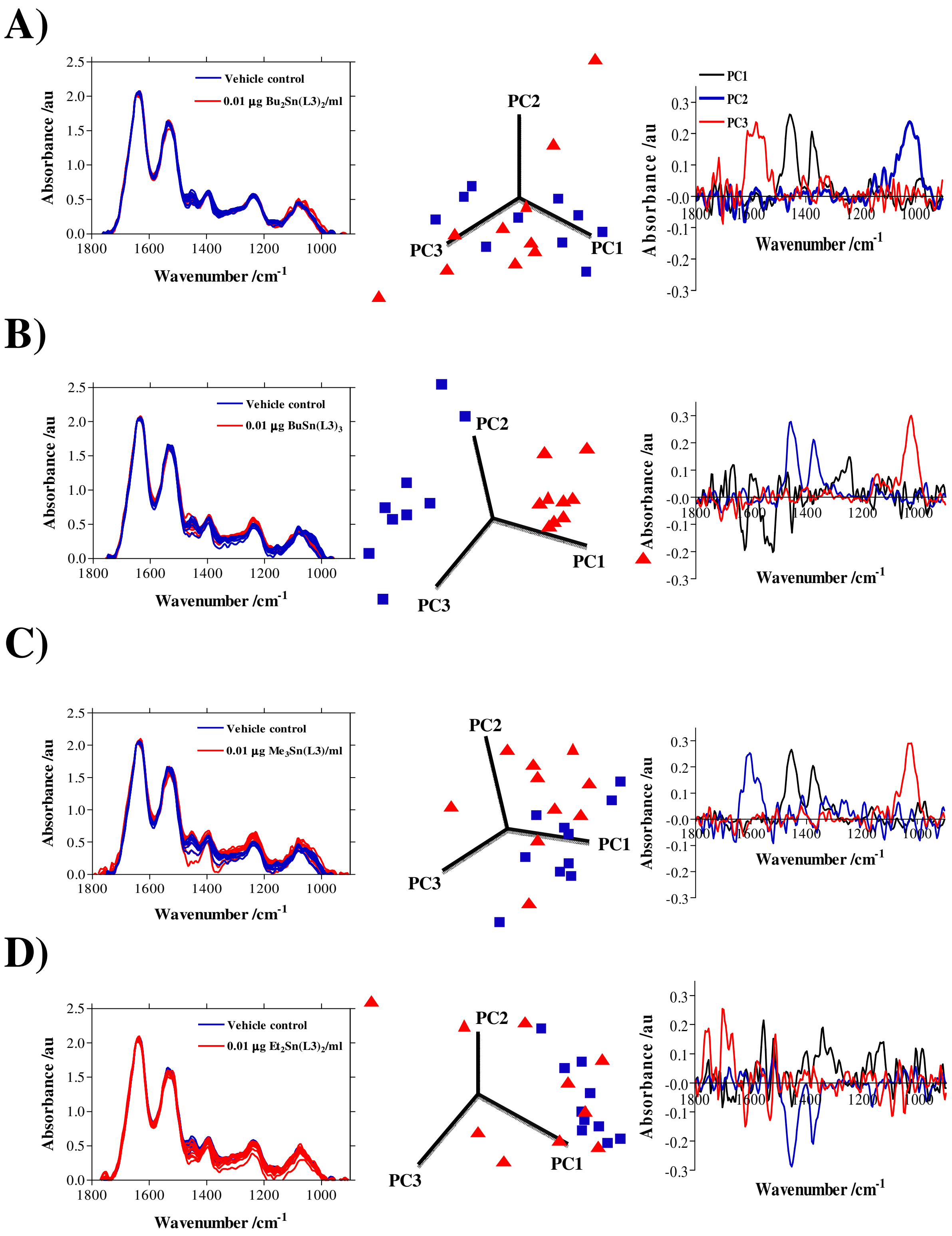

A)
Vehicle control
0.01 µg Bu$_2$Sn(L3)$_2$/ml
Absorbance /au
Wavenumber /cm^{-1}
PC1
PC2
PC3
B)
Vehicle control
0.01 µg BuSn(L3)$_3$
Absorbance /au
Wavenumber /cm^{-1}
PC1
PC2
PC3
C)
Vehicle control
0.01 µg Me$_3$Sn(L3)/ml
Absorbance /au
Wavenumber /cm^{-1}
PC1
PC2
PC3
D)
Vehicle control
0.01 µg Et$_2$Sn(L3)$_2$/ml
Absorbance /au
Wavenumber /cm^{-1}
PC1
PC2
PC3

ATR FTIR spectroscopy following 24-h exposure with 1.0 μg/ml

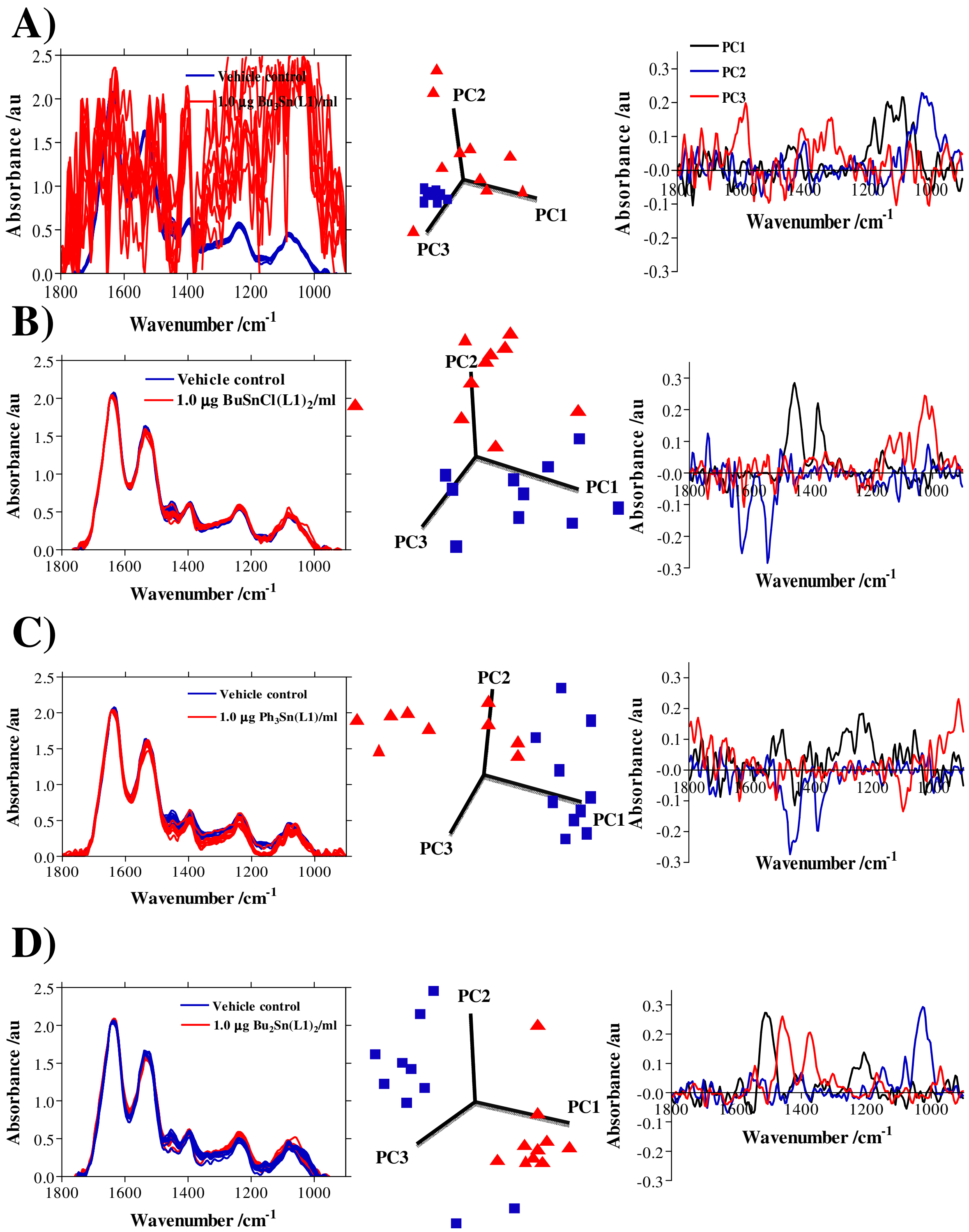

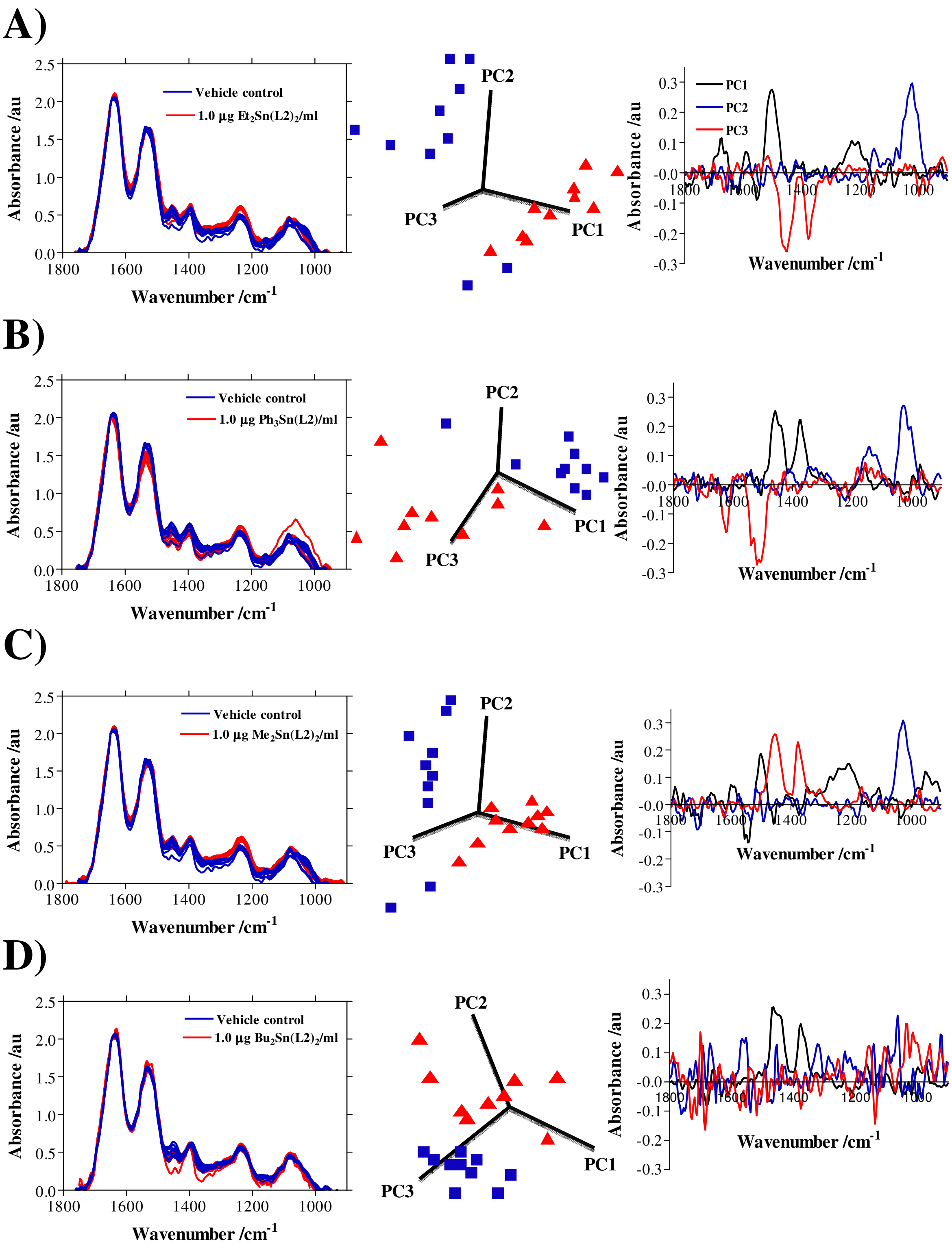
A)
Vehicle control
1.0 µg Et2Sn(L2)2/ml
Absorbance /au
Wavenumber /cm-1
PC1
PC2
PC3
B)
Vehicle control
1.0 µg Ph3Sn(L2)/ml
Absorbance /au
Wavenumber /cm-1
PC1
PC2
PC3
C)
Vehicle control
1.0 µg Me2Sn(L2)2/ml
Absorbance /au
Wavenumber /cm-1
PC1
PC2
PC3
D)
Vehicle control
1.0 µg Bu2Sn(L2)2/ml
Absorbance /au
Wavenumber /cm-1
PC1
PC2
PC3

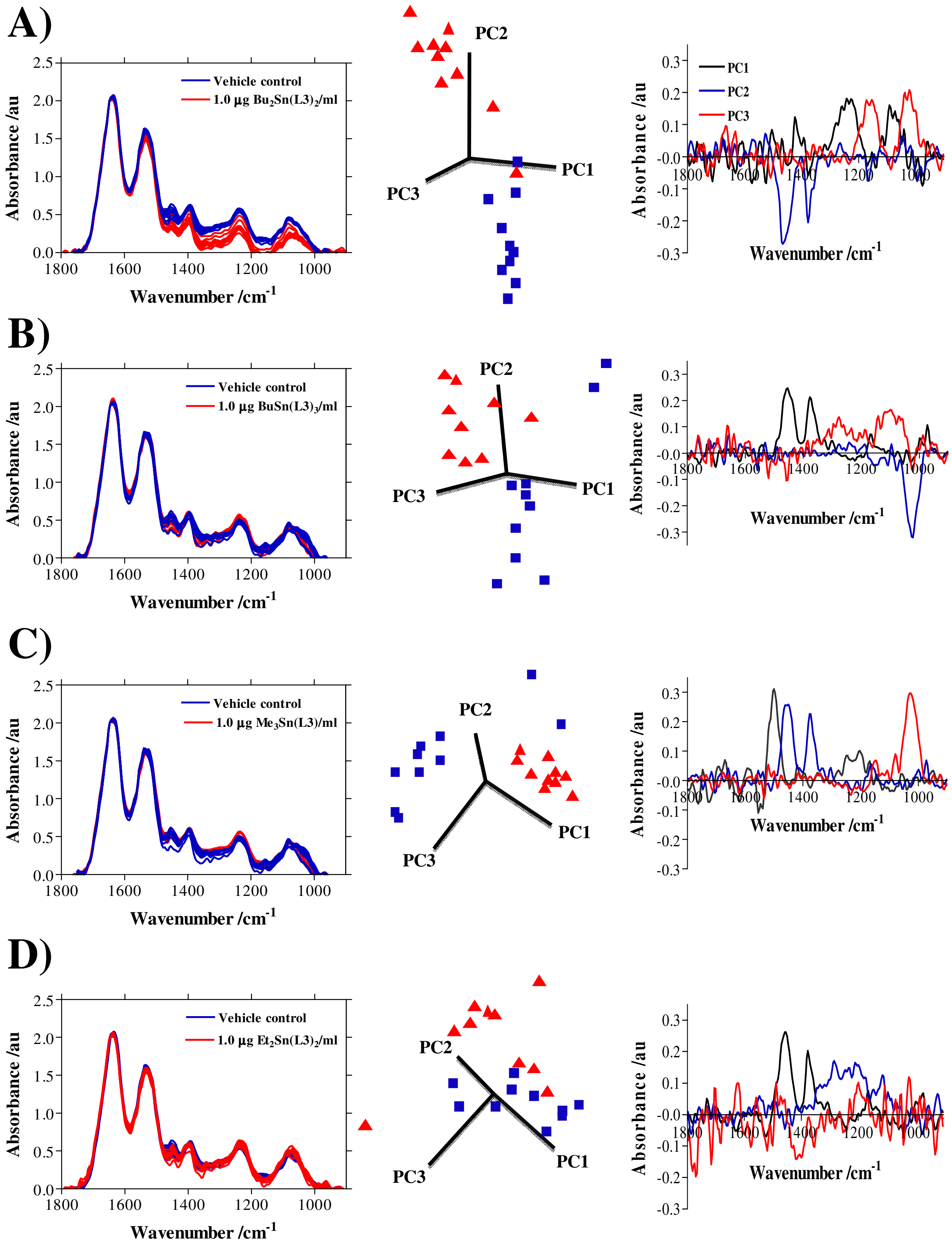

A)
Absorbance /au
Wavenumber /cm-1
Vehicle control
1.0 µg Bu2Sn(L3)2/ml
PC1
PC2
PC3
B)
Vehicle control
1.0 µg BuSn(L3)3/ml
C)
Vehicle control
1.0 µg Me3Sn(L3)/ml
D)
Vehicle control
1.0 µg Et2Sn(L3)2/ml
